# Positive rate‐dependent action potential prolongation by modulating potassium ion channels

**DOI:** 10.14814/phy2.15356

**Published:** 2022-06-24

**Authors:** Candido Cabo

**Affiliations:** ^1^ Department of Computer Systems New York City College of Technology Doctoral Program in Computer Science, Graduate Center City University of New York New York New York USA

**Keywords:** computer models, positive rate dependence, potassium ion channels

## Abstract

Pharmacological agents that prolong action potential duration (APD) to a larger extent at slow rates than at the fast excitation rates typical of ventricular tachycardia exhibit reverse rate dependence. Reverse rate dependence has been linked to the lack of efficacy of class III agents at preventing arrhythmias because the doses required to have an antiarrhythmic effect at fast rates may have pro‐arrhythmic effects at slow rates due to an excessive APD prolongation. In this report, we show that, in computer models of the ventricular action potential, APD prolongation by accelerating phase 2 repolarization (by increasing I_Ks_) and decelerating phase 3 repolarization (by blocking I_Kr_ and I_K1_) results in a robust positive rate dependence (i.e., larger APD prolongation at fast rates than at slow rates). In contrast, APD prolongation by blocking a specific potassium channel type results in reverse rate dependence or a moderate positive rate dependence. Interventions that result in a strong positive rate dependence tend to decrease the repolarization reserve because they require substantial I_K1_ block. However, limiting I_K1_ block to ~50% results in a strong positive rate dependence with moderate decrease in repolarization reserve. In conclusion, the use of a combination of I_Ks_ activators and I_Kr_ and I_K1_ blockers could result in APD prolongation that potentially maximizes antiarrhythmic effects (by maximizing APD prolongation at fast excitation rates) and minimizes pro‐arrhythmic effects (by minimizing APD prolongation at slow excitation rates).

## Graphical abstract

The use of a combination of IKs activators and IKr and IK1 blockers results in positive rate dependence and leads to APD prolongation that potentially maximizes anti‐arrhythmic effects (by maximizing APD prolongation at fast excitation rates) and minimizes pro‐arrhythmic effects (by minimizing APD prolongation at slow excitation rates).

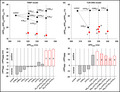

## 1 INTRODUCTION

Many cardiac arrhythmias have a reentrant mechanism, a pattern of excitation in which a wave rotates around an anatomical or functional obstacle (Peters et al., 2000). In order for the rotating wave to complete a reentrant cycle, the spatial extent of the reentrant wave (i.e., the wavelength, which is estimated as the product of myocardial conduction velocity times the tissue refractory period) has to be smaller than the perimeter of the anatomical or functional obstacle, leaving excitable tissue (i.e., an excitable gap) between the depolarizing head and the repolarizing tail of the reentrant wave. Class III pharmacological agents prolong the tissue refractory period by prolonging myocyte action potential duration (Tamargo et al., 2004; Vaughan, 1975). The rationale behind the antiarrhythmic action of class III agents is that an increase in refractory period would lead to an increase in the wavelength of the reentrant wave with the consequent decrease in the excitable gap, causing the depolarizing head of the reentrant wave to run into refractory tissue extinguishing the arrhythmia.

However, clinical trials have shown that class III antiarrhythmic drugs are not effective in preventing initiation and maintenance of reentrant arrhythmias in post‐myocardial infarction patients (Bloch‐Thomsen, 1998; Køber et al., 2000; Waldo et al., 1996). As it turns out, Class III agents prolong action potential duration to a greater extent at slow heart rates (e.g., during sinus rhythm) than at fast rates (e.g., during ventricular tachycardia), a property known as reverse rate dependence. This inability of class III agents to prolong action potential duration at the fast excitation rates typical of ventricular tachycardia has been linked to the lack of efficacy of class III agents at preventing arrhythmias (Hondeghem & Snyders, 1990). Moreover, agents that prolong the action potential at slow rates may result in drug‐induced LQT syndrome, trigger early afterdepolarizations, and have a pro‐arrhythmic effect (Kannankeril et al., 2010). Ideally, class III agents should prolong the action potential at fast rates with a minimal prolongation at slow rates, that is, they should exhibit a positive rate dependence, which is the opposite of a reverse rate dependence (Hondeghem & Snyders, 1990; Winter & Shattock, 2015). Positive rate dependence is also referred to in the literature as forward rate dependence (Cummins et al., 2014).

It has been postulated that reverse rate dependence is an intrinsic property of ventricular myocardium (Banyasz et al., 2009). However, there is evidence that some pharmacological agents like amiodarone that prolong the action potential by blocking sodium and potassium channels have an attenuated reverse rate dependence response when compared to agents that block selectively a unique ion channel type (Dorian & Newman, 2000; Hondeghem & Snyders, 1990). We hypothesized that a prolongation of the action potential by modulating several potassium channels (instead of just a unique potassium channel type) may result in positive rate dependence or at least in an attenuated reverse rate dependence. We used computational models of the ventricular action potential in combination with optimization algorithms to investigate which interventions may result in prolongation of the action potential with a positive rate dependence (or an attenuated reverse rate dependence), and to further understand how the morphology of the action potential relates to its rate dependence.

## 2 METHODS

### 2.1 Computer models of the action potential

We simulated the cardiac action potential using the TNNP (ten Tusscher et al., 2004) and the ToR‐ORd (Tomek et al., 2019) models of a human ventricular epicardial cell. The models are publicly available and were downloaded from the CellML repository (www.cellml.org). We investigated the rate dependence of the action potential models by modulating the maximum conductance of the slow delayed rectifier potassium current (G_Ks_) between 0 and 2× its standard value in the TNNP model, and between 0 and 10× its standard value in the ToR‐ORd model. In both models the maximum conductance of the rapid delayed rectifier potassium current (G_Kr_) was modulated between 0 and 2× its standard value, and the maximum conductance of the inward rectifier potassium current (G_K1_) was modulated between 0.2× and 2× its standard value. All simulations were performed in a single cell. Action potentials were initiated with a depolarizing current with a strength 1.5× the stimulation threshold. We report measurements on action potentials that were calculated after 16 min of stimulation. APD values after 16 min of stimulation are within 1 ms of the APD values at steady‐state.

### 2.2 Calculation of average total ionic current from the action potential shape

In a cell membrane, the total membrane current (I_m_) is the sum of the ionic current and the capacitive current:

$$I_{m}=I_{\mathrm{ion}}+C_{m}\;\frac{dV_{m}}{\mathrm{dt}}$$

where *C*_*m*_ is the membrane specific capacitance (in μF/cm^2^) and V_m_ is the transmembrane voltage (in mV). For a single cell, there is no axial current, which means that I_m_ *= 0*, therefore:

$$I_{\mathrm{ion}}=-C_{m}\;\frac{dV_{m}}{\mathrm{dt}}$$

Integrating the previous equation from any two points in the action potential, $V_{m,t_{1}}$ (the membrane potential at *t*_1_) and $V_{m,t_{2}}$ (the membrane potential at *t*_2_), where *t*_2_ > *t*_1_, we obtain:

$$\begin{array}{c} \int_{t_{1}}^{t_{2}}I_{\mathrm{ion}}\mathrm{dt}=-C_{m}\int_{t_{1}}^{t_{2}}\frac{dV_{m}}{\mathrm{dt}}\mathrm{dt}\\ \;=-C_{m}\left(V_{m,t_{2}}-V_{m,t_{1}}\right)=C_{m}\left(V_{m,t_{1}}-V_{m,t_{2}}\right) \end{array}$$

Using the mean value theorem, we can write:

$$\int_{t_{1}}^{t_{2}}I_{\mathrm{ion}}\mathrm{dt}=\bar{I_{\mathrm{ion}}}\left(t_{2}-t_{1}\right)=-C_{m}\left(V_{m,t_{2}}-V_{m,t_{1}}\right)$$

where $\bar{I_{\mathrm{ion}}}$ is the average of I_ion_ in the interval [*t*_1_, *t*_2_]. Ionic currents measured experimentally are typically normalized to the cell capacitance. Therefore, normalizing the previous expression to *C*_*m*_, we obtain:

$$\left(\frac{\bar{I_{\mathrm{ion}}}}{C_{m}}\right)=\frac{-\left(V_{m,t_{2}}-V_{m,t_{1}}\right)}{\left(t_{2}-t_{1}\right)}$$

where now the current is in (pA/pF). This expression shows that the average I_ion_ ($\bar{I_{\mathrm{ion}}}$) between any two points in the action potential is the slope of the line joining those two points.

### 2.3 Action potential features

**FIGURE 1:**
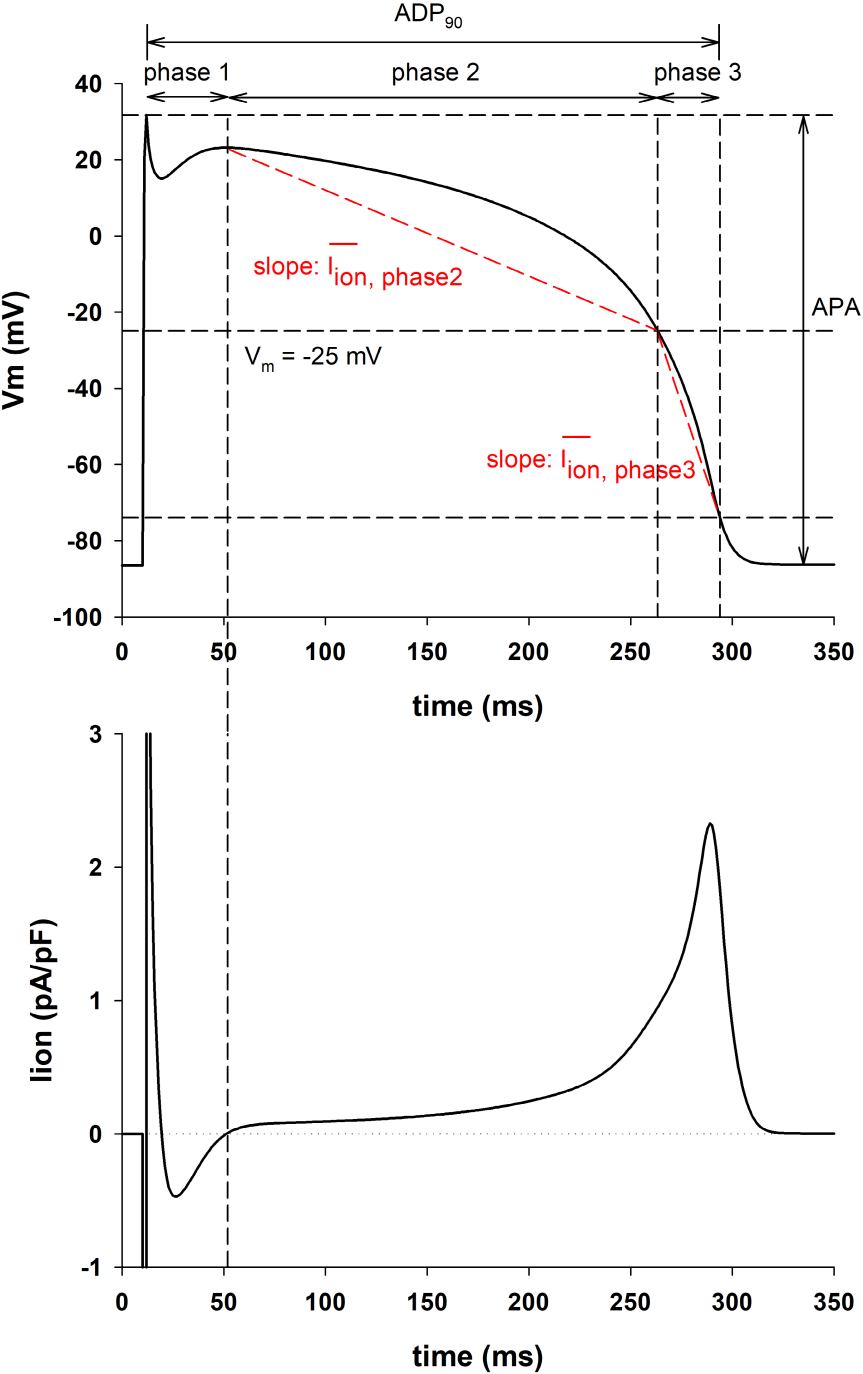
Features of the action potential. Top: Action potential calculated with the TNNP model. The figure shows the features of the action potential used in this study: Action potential amplitude (APA); action potential duration at 90% repolarization (APD_90_); duration of phase 1, phase 2, and phase 3; average ionic current during phase 2 (I_ion, phase2_) and during phase 3 (I_ion, phase3_). Bottom: Total ionic current during the repolarization of the action potential. The vertical dashed line shows the time when I_ion_ changes from negative to positive indicating the end of phase 1 and the beginning of phase 2 repolarization.

To quantify and compare different action potential shapes we extracted the following features from the action potential (Figure 1, top): action potential amplitude (APA); action potential duration at 90% repolarization (APD_90_); duration of phase 1, phase 2, and phase 3; average ionic current during phase 2 (I_ion, phase2_) and during phase 3 (I_ion, phase3_); ratio of I_ion, phase3_ and I_ion, phase2_; and the area under the action potential (AUAP) between the time of depolarization and the time of repolarization to APD_90_ normalized to APD_90_xAPA.

Phase 1 begins at the time of action potential depolarization and ends at the time repolarization starts, which is when the total ionic current (Figure 1, bottom) becomes positive (dashed vertical line in Figure 1). Phase 2 starts when phase 1 ends, and it ends when I_K1_ raises to 10% of its peak (we could not find in the literature a clear quantitative marker of the end of phase 2). In the TNNP model the end of phase 2 occurs when the membrane repolarizes to −25 mV. In the ToR‐ORd model the end of phase 2 occurs when the membrane repolarizes to −34 mV. Phase 3 starts at the end of phase 2, and it ends when the action potential repolarizes from its maximum depolarization potential to 90% of the action potential amplitude (APA).

The average ionic current during phase 2 (I_ion, phase2_ in Figure 1) is the slope of the line joining the *V*_m_ of the action potential where phase 2 starts and the point of the action potential where phase 2 ends (dashed red line in Figure 1). The same applies to the average ionic current during phase 3 (I_ion, phase3_ in Figure 1).

### 2.4 Estimation of the repolarization reserve

Interventions that prolong APD may increase the risk of arrhythmias as a result of early afterdepolarizations. The concept of a repolarization reserve was developed to assess that pro‐arrhythmic risk (Roden, 1998). In this report, we estimated the repolarization reserve of a baseline action potential by quantifying the prolongation of the APD upon application of a constant depolarizing current of −0.1pA/pF during the action potential (Varro & Baczko, 2011). This can be done experimentally for example by increasing the persistent sodium current with veratrine and anemonia sulcata toxin (ATX II) (Varro & Baczko, 2011). With that protocol, a larger prolongation of the APD with respect to the baseline APD implies a smaller repolarization reserve and a higher risk of triggered arrhythmias.

### 2.5 Optimization algorithm

When using specific potassium channel blockers to prolong the action potential, the prolongation of the action potential (with respect to control) at long cycle lengths (ΔAPD_long_) is generally larger than the prolongation of the action potential at short cycle lengths (ΔAPD_short_), resulting in reverse use dependence (ΔAPD_long_ > ΔAPD_short_). We used the particle swarm optimization (PSO) algorithm (Kennedy & Eberhart, 1995) to find the optimal combination of maximum conductance of I_Ks_, I_Kr_, and I_K1_ to minimize the difference between ΔAPD_long_ and ΔAPD_short_ while achieving a given APD prolongation. We used an implementation of the PSO algorithm publicly available in the Github repository (https://github.com/kkentzo/pso). Minimizing ΔAPD_long_−ΔAPD_short_ should result in an attenuation of reverse rate dependence or achieving positive rate dependence if ΔAPD_long_ < ΔAPD_short_ for a given APD. In the simulations presented here, for both the TNNP and ToR‐ORd model, the long cycle length was BCL = 3000 ms, and the short cycle length was BCL = 400 ms. The input of the PSO optimization algorithm was the APD goal and the allowed range of variation (minimum and maximum values) of G_Ks_, G_Kr_, and G_K1_. The range of variation for G_Ks_ was between 0 and 2× the standard value of G_Ks_ for the TNNP model, and between 0 and 10× the standard value for the ToR‐ORd model. The range of variation for G_Kr_ was between 0 and 2× the standard value of G_Kr_ for both models. The minimum value of G_K1_ was 0.2×, 0.5×, 0.7×, and 1× the standard value depending on the PSO optimization protocol. The maximum value of G_K1_ was always 2× the standard value. The output of the algorithm was the optimal values of G_Ks_, G_Kr_, and G_K1_ that minimize ΔAPD_long_−ΔAPD_short_ (the more negative this number the stronger the positive rate‐dependent response) for a given APD goal.

## 3 RESULTS

### 3.1 Prolongation of the action potential with reverse and positive rate dependence

**FIGURE 2:**
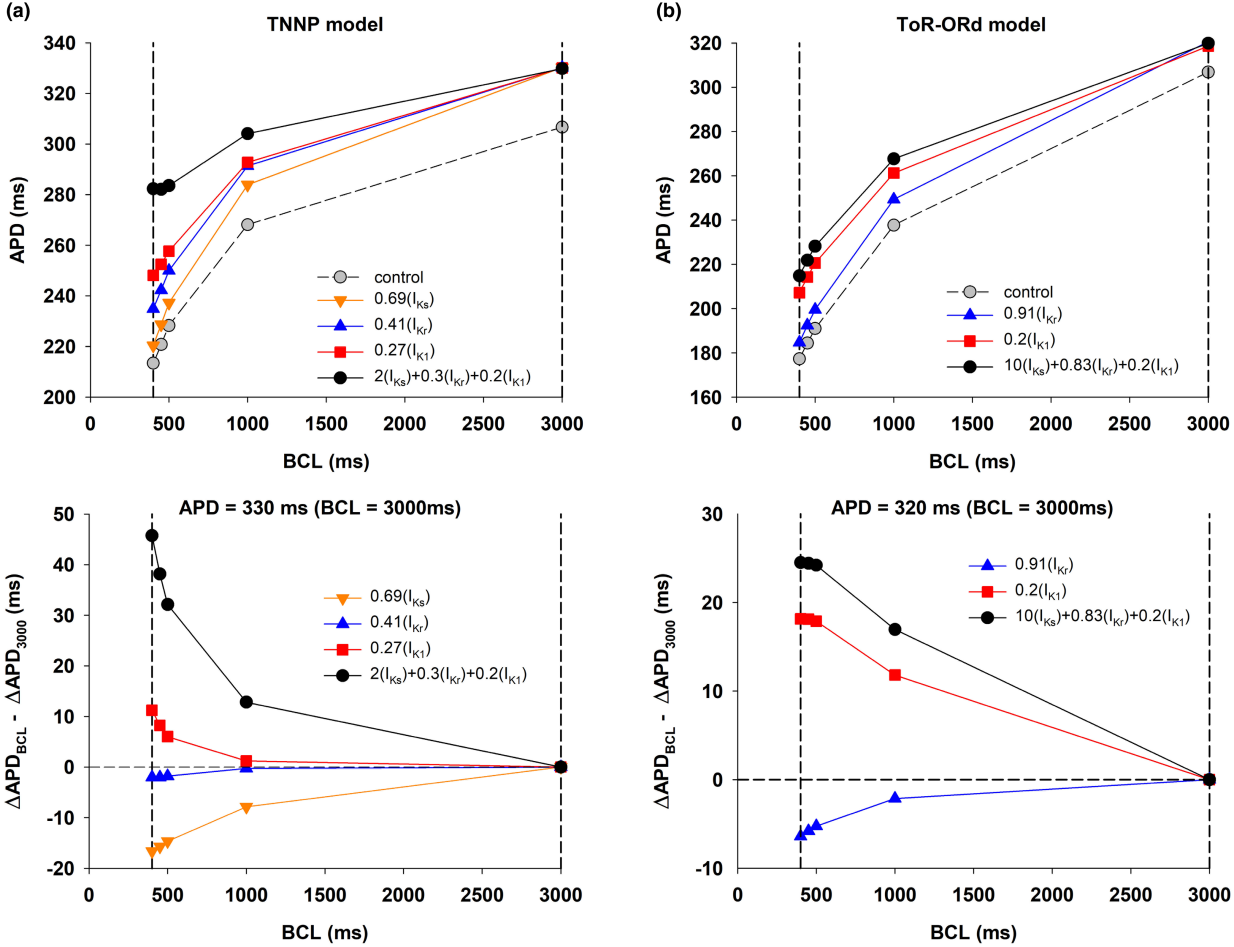
Rate dependence of APD prolongation with the TNNP and ToR‐ORd models. Panel a (top): APD rate dependence during control (gray circles), and for four interventions that prolonged the control APD to 330 ms when BCL = 3000 ms, using the TNNP model. The vertical dashed lines indicate BCL = 400 and 3000 ms, respectively. Panel a (bottom): APD prolongation at the different BCLs compared with APD prolongation at BCL = 3000 ms for the four interventions in Panel a (top). Positive values indicate a positive rate dependence and a negative value indicates reverse rate dependence. Panel b (top): APD rate dependence during control (gray circles), and for three interventions that prolonged the control APD to 320 ms at BCL = 3000 ms, using the ToR‐ORd model. The vertical dashed lines indicate BCL = 400 and 3000 ms, respectively. Panel b (bottom): APD prolongation at the different BCLs compared with APD prolongation at BCL = 3000 ms for the three interventions in Panel a (top). Positive values indicate a positive rate dependence and a negative value indicates reverse rate dependence. See text for detailed description.

Figure 2a (top) shows the APD rate dependence during control (gray circles), and for four interventions that prolonged the control APD to 330 ms at BCL = 3000 ms, using the TNNP model. Note that while APD prolongation at BCL = 3000 ms (right vertical dashed line) is the same for all interventions, APD prolongation for shorter BCLs was markedly different. Figure 2a (bottom) shows how APD prolongation at a given BCL compares to APD prolongation at BCL = 3000 ms for the four interventions. A positive value indicates that APD prolongation at a given BCL is larger that APD prolongation at 3000 ms, and shows a positive rate dependence; a negative value indicates a reverse rate dependence. APD prolongation by blocking I_Ks_ by 31% (0.69[I_Ks_], ochre triangles down) or by blocking I_Kr_ by 59% (0.41[I_Kr_], blue triangles up) show a negative rate dependence. In contrast, 73% block of I_K1_ (0.27[I_K1_], red squares) shows a moderate positive rate dependence. The optimal combination of potassium channel activators (of I_Ks_) and blockers (of I_Kr_ and I_K1_) found by the PSO algorithm (2(I_Ks_) + 0.3(I_Kr_) + 0.2(I_K1_), black circles) shows a robust positive rate‐dependent response.

Figure 2b shows similar results for interventions that prolong APD to 320 ms using the ToR‐ORd model. As with the TNNP model, while the prolongation of APD at BCL = 3000 ms (right vertical dashed line) is the same for all interventions, APD prolongation for shorter BCLs was markedly different (Figure 2b, top). As with the TNNP model, APD prolongation by blocking I_Kr_ (0.91(I_Kr_), blue triangles up) resulted in reverse rate dependence (Figure 2b, bottom), while APD prolongation by blocking I_K1_ (0.2(I_K1_), red squares) resulted in positive rate dependence. The optimal combination of potassium channel activators (of I_Ks_) and blockers (of I_Kr_ and I_K1_) identified by the PSO algorithm (10(I_Ks_) + 0.83(I_Kr_) + 0.2(I_K1_), black circles) again produced a stronger positive rate dependence than the intervention blocking a unique potassium channel type (I_K1_). We did not evaluate the rate dependence characteristics of I_Ks_ because I_Ks_ is very small in the ToR‐ORd model, and prolongation of APD by complete block of I_Ks_ is negligible (~1% of the control APD).

**FIGURE 3:**
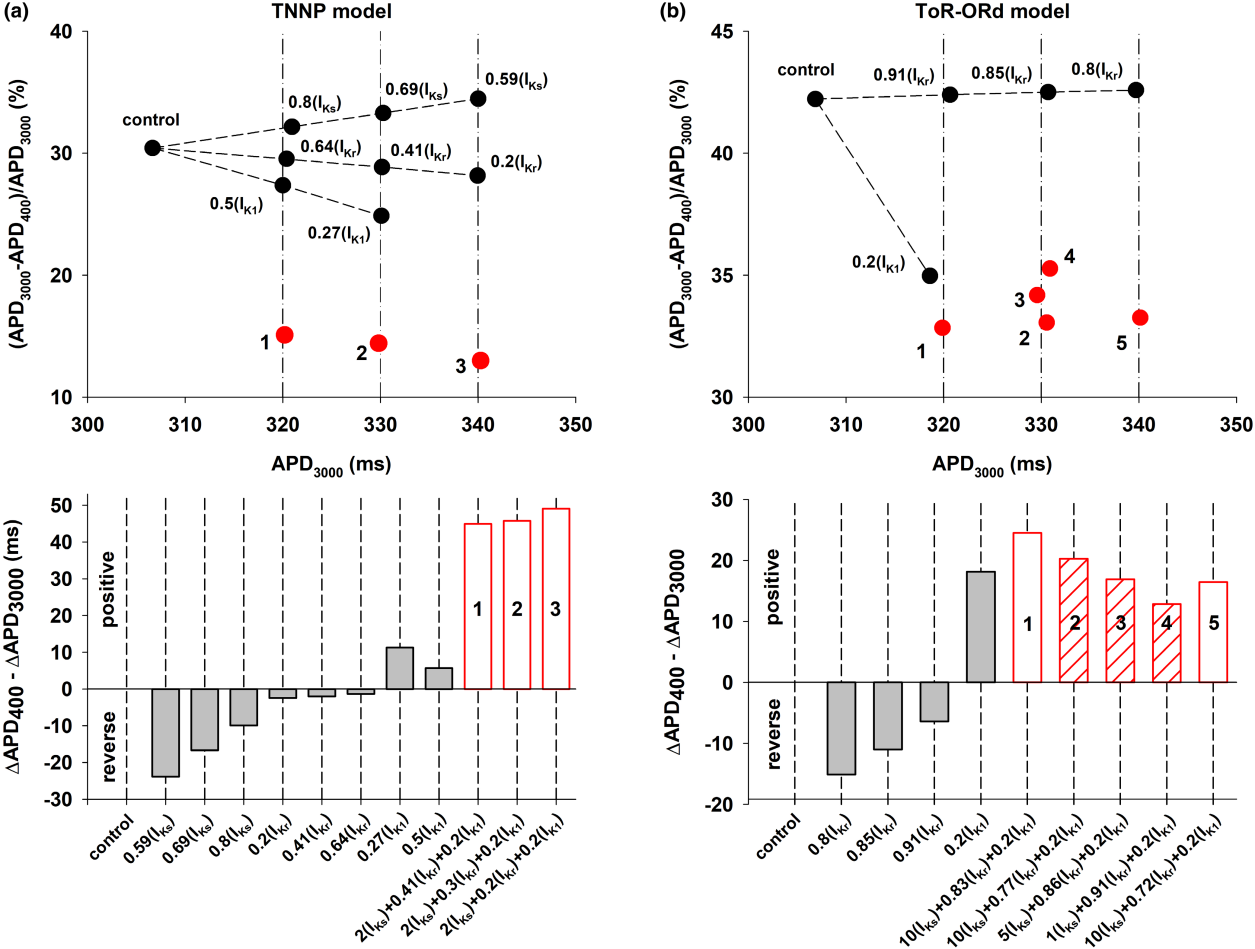
Panel a (top): Percentage APD shortening between BCL = 3000 ms and 400 ms, for different interventions prolonging APD to 320, 330, and 340 ms when BCL = 3000 ms (vertical dashed‐dot lines) by blocking a unique type of potassium channel (black circles), and by activation/block of several potassium channels identified by POS optimization (red circles), using the TNNP model. Panel a (bottom): Difference between the prolongation of APD with BCL = 400 ms and BCL = 3000 ms for the interventions in panel a (top). Negative values indicate reverse rate dependence and positive values indicates positive rate dependence. White bars with red edges labeled 1, 2, 3 indicate interventions that modulate several potassium channels and correspond to the red circles in panel a (top). Gray bars indicate interventions that block a unique potassium channel and correspond to the black circles in panel a (top). Panel b (top) percentage APD shortening between BCL = 3000 ms and 400 ms for interventions that prolong APD to 320, 330, and 340 ms when BCL = 3000 ms (vertical dashed‐dot lines), using the ToR‐ORd model. Black and red circles and labels have the same meaning as in Panel a (top). Panel b (bottom): Difference between the prolongation of APD with BCL = 400 ms and BCL = 3000 ms for the interventions in panel b (top). Gray and white bars with red edges have the same meaning as in Panel a (bottom). See text for detailed description.

Figure 3a (top) summarizes the percentage APD shortening between BCL = 3000 ms and 400 ms (defined as [APD_BCL = 3000_ – APD_BCL = 400_] / APD_BCL = 3000_) for different interventions that prolong the control APD to 320, 330, and 340 ms, using the TNNP model (vertical dashed‐dot lines in Figure 3, top). Black circles represent interventions that prolong ADP by modulating a unique potassium channel type; red circles (labeled 1, 2, 3) represent interventions identified by the POS optimization algorithm to prolong APD maximizing positive rate dependence. Interventions along the central vertical dashed‐dot line (APD = 330 ms) correspond to the interventions shown in Figure 2a. Note that the percentage APD shortening for interventions identified by POS optimization (red circles) are considerably smaller than for interventions that block a unique potassium channel type (black circles).

When APD prolongation (with respect to control) at BCL = 3000 ms (ΔAPD_BCL = 3000_) is larger than APD prolongation (with respect to control) at BCL = 400 ms (ΔAPD_BCL = 400_) that is, when ΔAPD_BCL = 400_ – ΔAPD_BCL = 3000_ is negative, the intervention results in reverse rate dependence; otherwise, the intervention results in positive rate dependence. Figure 3a (bottom) shows whether the interventions in Figure 3a (top) exhibit a reverse (negative value in Figure 3a, bottom) or positive rate dependence (positive value in Figure 3a, bottom). Prolongation of APD by blocking independently I_Ks_, I_Kr_, or I_K1_ results in either reverse rate dependence (for 0.59(I_Ks_), 0.69(I_Ks_), 0.8(I_Ks_), 0.2(I_Kr_), 0.41(I_Kr_), 0.64(I_Kr_)) or a relatively small positive rate dependence (0.27(I_K1_) and 0.5(I_K1_)) (gray bars in Figure 3a, bottom). However, interventions that prolong the action potential by enhancing I_Ks_ and blocking I_Kr_ and I_K1_, result in a robust positive rate dependence response (white bars with red edges labeled 1, 2, and 3 in Figure 3a, bottom).

Figure 3b shows results of computations with the ToR‐ORd model, which are similar to those obtained with the TNNP model (Figure 3a). Interventions along the left vertical dashed‐dot line (APD = 320 ms, Figure 3b, top) correspond to the interventions shown in Figure 2b. Note that the percentage APD shortening for interventions that block I_K1_ or the interventions identified by POS optimization (red circles) are smaller than for interventions that block I_Kr_. Figure 3b (bottom) shows whether the interventions resulted in a reverse or positive rate dependence. As it happened with the TNNP model, interventions that block I_Kr_ resulted in reverse rate dependence and interventions that blocked I_K1_ resulted in positive rate dependence. As we mentioned earlier, we did not evaluate the rate dependence characteristics of I_Ks_ because prolongation of APD by complete block of I_Ks_ is negligible in the ToR‐ORd model. Likewise, interventions that prolong the action potential by enhancing I_Ks_ and blocking I_Kr_ and I_K1_, result in a robust positive rate dependence response (white bars with red edges labeled 1, 2, 3, 4, and 5 in Figure 3b, bottom). The bars with hatched red lines, labeled as 2, 3, and 4 in Figure 3b (bottom), illustrate that the larger the I_Ks_ enhancement the larger the positive rate‐dependent response is, showing the importance of enhancing I_Ks_ (in combination with blocking I_Kr_ and I_K1_) to obtain a positive rate‐dependent APD prolongation, even in a model of the action potential with a small I_Ks_ current.

**FIGURE 4:**
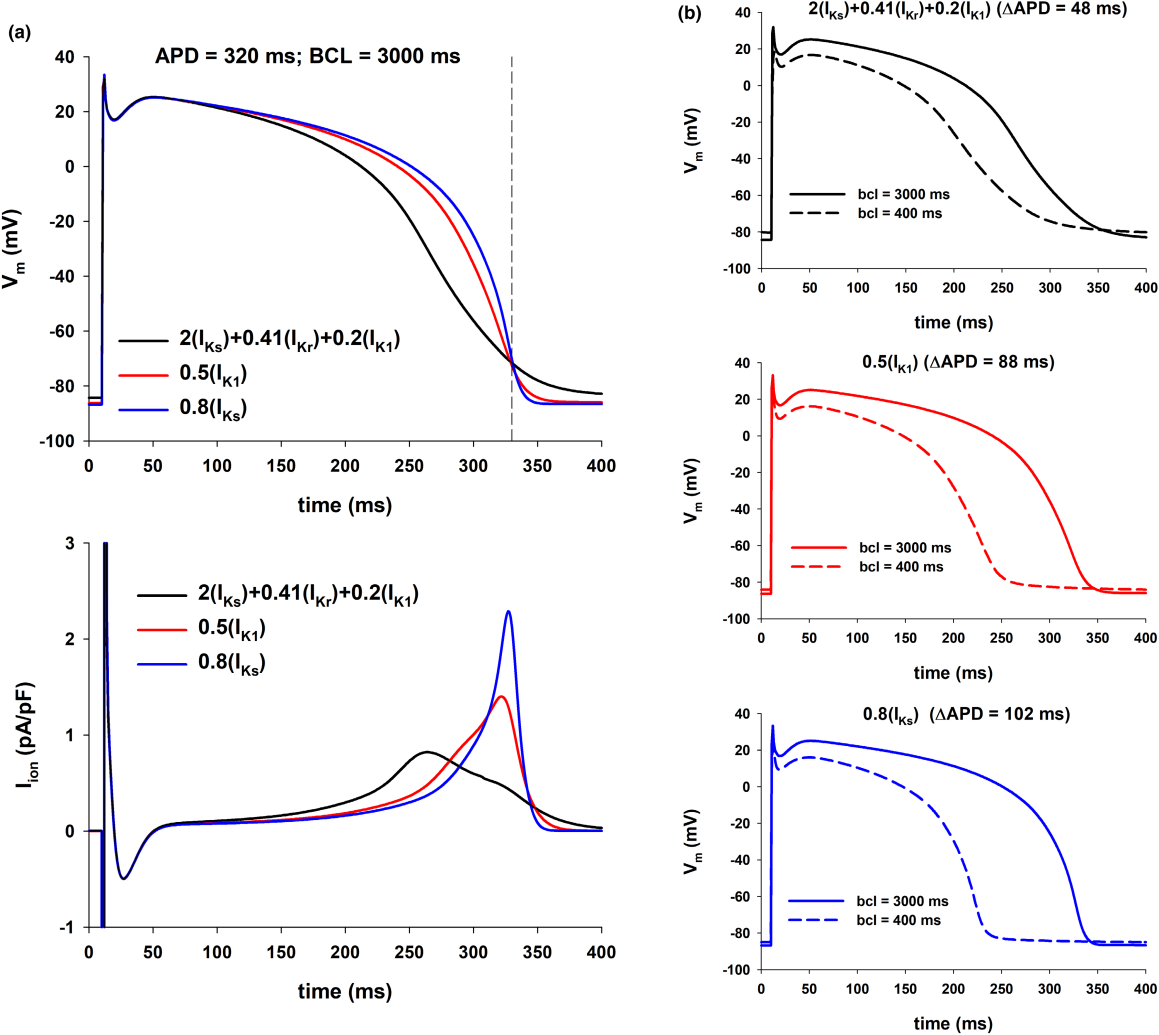
Influence of the action potential shape on its rate dependence (TNNP model). Panel a: Action potentials for different interventions that prolong the action potential to 320 ms for BCL = 3000 ms (top) with the corresponding total ionic currents (bottom) (vertical dashed‐dot line through red circle labeled as 1 in Figure 3a, top). Panel b: Action potentials for BCL = 3000 (solid line) and 400 ms (dashed line) for the interventions in panel a. The action potential with a faster phase 2 repolarization and slower phase 3 repolarization (2(I_Ks_) + 0.41(I_Kr_) + 0.2(I_K1_)) results is a smaller APD shortening with increasing stimulation rate than the other two: 48 ms versus 88 ms for 0.5(I_K1_) and 102 ms for 0.8(I_Ks_).

**FIGURE 5:**
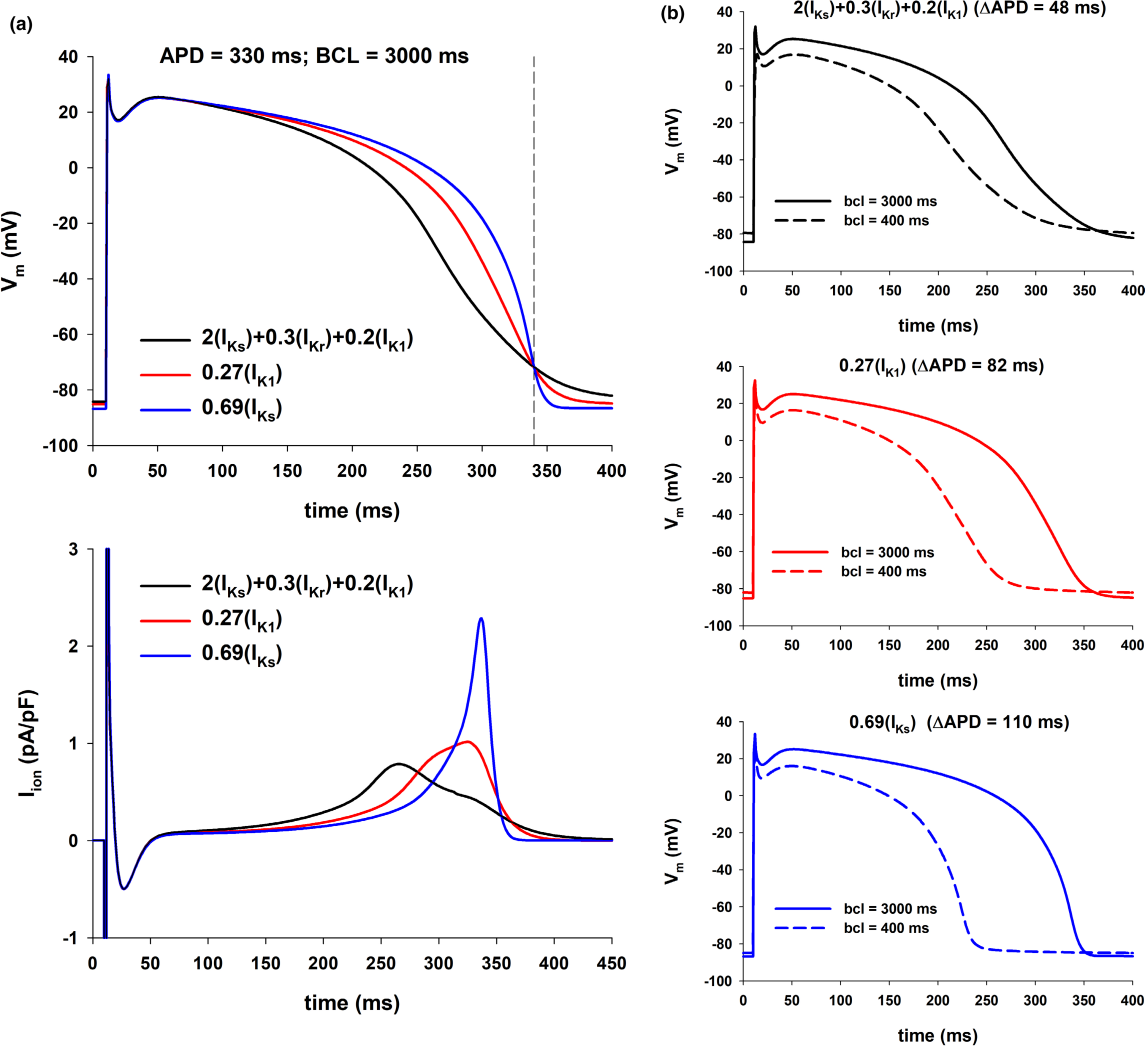
Influence of the action potential shape on its rate dependence (TNNP model). The figure shows action potentials and ionic currents resulting from interventions that prolong the action potential to 330 ms for BCL = 3000 ms (vertical dashed‐dot line through red circle labeled as 2 in Figure 3a, top). The figure uses the same format as Figure 4.

The results in Figures 2 and 3 suggest that the TNNP model can produce a more robust positive rate dependence than that of the ToR‐ORd model, possibly as a result of a larger I_Ks_ current in control. For example, ΔAPD_BCL = 400_ – ΔAPD_BCL = 3000_ is about 50 ms for the TNNP model (white bars with red edges, Figure 3a, bottom), but about 13–25 ms for the ToR‐ORd model (white bars with red edges, Figure 3b, bottom). However, for both models, for different amounts of APD prolongation, increasing I_Ks_ and blocking I_Kr_ and I_K1_ (interventions labeled 1, 2, 3 in Figures 3a and 1, 2, 3, 4, 5 in 3b) result in a robust positive rate dependence, which cannot be achieved by blocking a unique potassium channel type. Given that consistency, in what follows we present results of computations that use the TNNP model.

### 3.2 Action potential shape influences its rate dependency

We then investigated how different features of the action potential shape influence its rate dependency. Figure 4a (top) shows the action potentials for three of the interventions that resulted in APD = 320 ms with BCL = 3000 ms (vertical dashed‐dot line through red circle labeled 1 in Figure 3a, top), using the TNNP model. The action potentials are superimposed to make it easier to compare their shapes. Figure 4a (bottom) shows the corresponding total ion currents during the action potential. Note that during the first 100 ms (phase 1 and beginning of phase 2) of the action potential there are no differences in shape, but the shape during phase 2 and phase 3 is markedly different for the three action potentials shown. The 2(I_Ks_) + 0.41(I_Kr_) + 0.2(I_K1_) action potential (black line in Figure 4a, top), repolarizes faster than the other two during phase 2, but slower during phase 3. This is also shown in Figure 4a (bottom) where the repolarizing current between 150 and 275 ms is largest for the black line action potential, but during the final phase of repolarization (300 ms to 350 ms), the repolarizing current is largest for the 0.8(I_Ks_) action potential (blue line in Figure 4a).

Figure 4b shows rate‐dependent changes for the three action potentials in Figure 4a: solid action potentials were calculated with BCL = 3000 ms; dashed action potentials were calculated with BCL = 400 ms. The figure shows that the action potential that has a more pronounced phase 2 repolarization and less pronounced phase 3 repolarization (2(I_Ks_) + 0.41(I_Kr_) + 0.2(I_K1_), Figure 4b, top) exhibits a smaller APD shortening (48 ms) with an increased stimulation rate than the other two action potentials (88 ms for 0.5(I_K1_) and 102 ms for 0.8(I_Ks_)).

**FIGURE 6:**
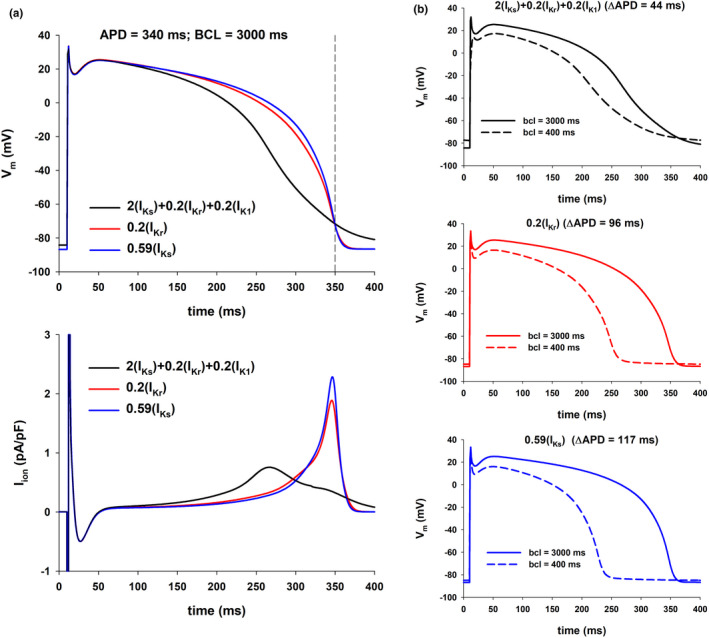
Influence of the action potential shape on its rate dependence (TNNP model). The figure shows action potentials and ionic currents resulting from interventions that prolong the action potential to 340 ms for BCL = 3000 ms (vertical dashed‐dot line through red circle labeled as 3 in Figure 3a, top). The figure uses the same format as Figure 4.

Figures 5 and 6 shows similar results for APD = 330 ms and APD = 340 ms with BCL = 3000 ms (vertical dashed‐dot lines through red circles labeled 2 and 3 in Figure 3a, top). As in Figure 4, action potentials with a more pronounced repolarization during phase 2 and a less pronounced repolarization during phase 3 (resulting in a triangulation of the action potential) have a smaller APD percentage shortening with an increased stimulation rate, and result in a robust positive rate dependence (Figure 3a, bottom).

### 3.3 Action potential features and APD rate dependency

**FIGURE 7:**
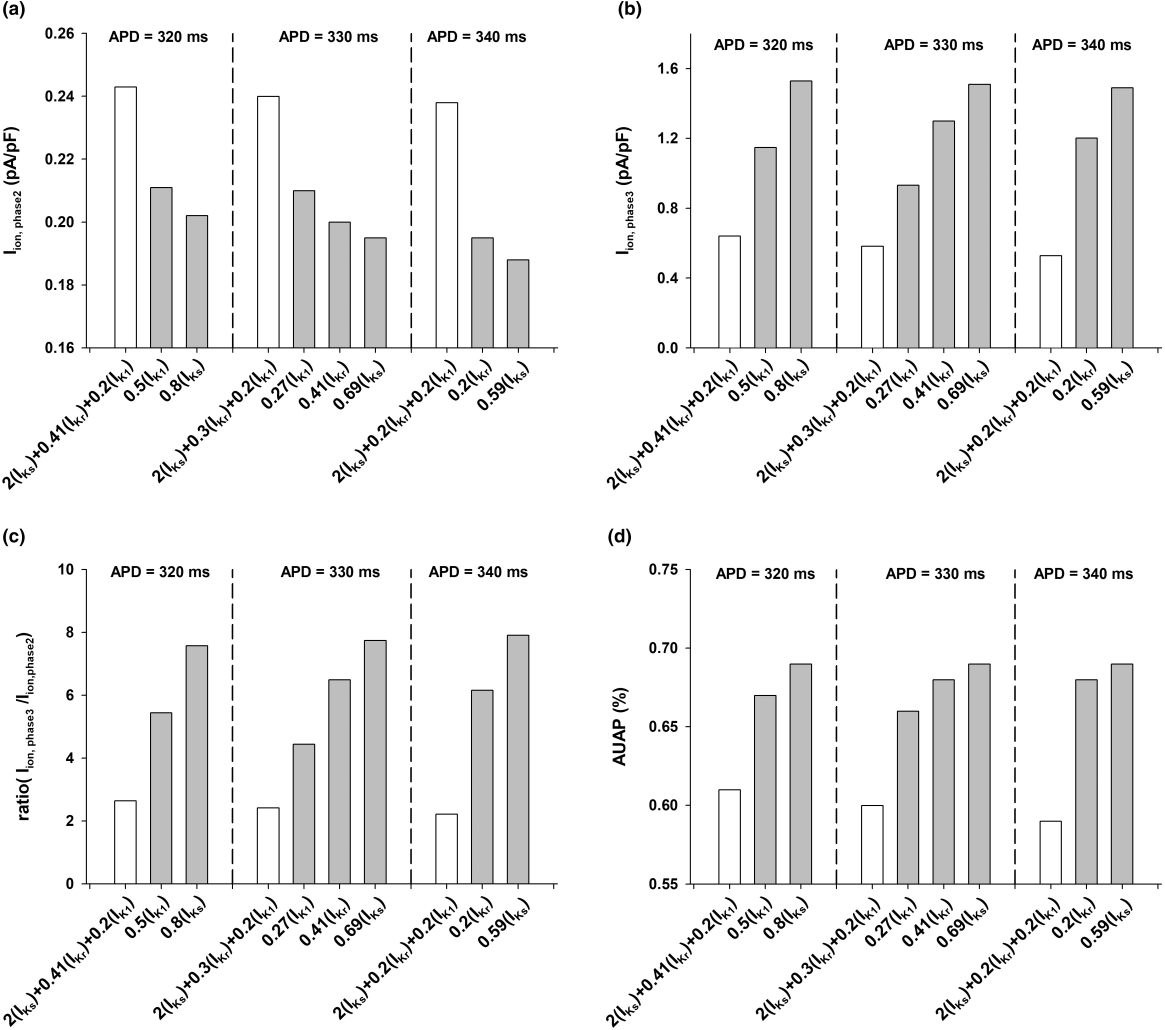
Features extracted from action potentials corresponding for the interventions in Figures 4 (APD = 320 ms), 5 (APD = 330 ms), and 6 (APD = 340 ms) for BCL = 3000 ms (TNNP model). White vertical bars represent interventions identified by the POS optimization algorithm, which involve the modulation of several potassium channels; gray vertical bars represent interventions involving modulation of one potassium channel. The features show the average ionic current during phase 2 (panel a), the average ionic current during phase 3 (panel b), the ratio of the average currents during phase 3 and phase 2 (panel c), and the area under the action potential (AUAP) normalized to APA and APD_90_ (panel d). The interventions identified by the POS algorithm result in a more triangularized action potential (smaller ratio in panel c and a smaller AUAP in panel d), with a positive rate dependence (Figure 3a, bottom).

Figure 7 shows four features extracted from the action potentials in Figures 4, 5, and 6: average ionic current during phase 2, I_ion, phase2_ (panel a), average ionic during phase 3, I_ion, phase3_ (panel b), the ratio of the averages of I_ion, phase3_ and I_ion, phase2_ (panel c), and the area under the action potential (AUAP) between the time of depolarization and the time of repolarization to APD_90_ normalized to APD_90_xAPA (panel d). The white vertical bars represent features from action potentials found by the POS optimizer to prolong APD to 320, 330, and 340 ms with a maximal positive rate dependence response. Those action potentials result from the combined modulation of I_Ks_, I_Kr_, and I_K1_. The gray vertical bars represent action potentials that prolong the APD to 320, 330, and 340 ms by blocking a unique potassium channel type and that result in reverse or moderate positive rate dependence. Action potentials with a positive rate dependence are associated with a larger average ionic current during phase 2 (Figure 7a), a smaller average ionic current during phase 3 (Figure 7b), resulting in a smaller ratio of average ionic currents during phase 3 and phase 2 triangularizing the action potential (Figure 7c), and a smaller area under the action potential (Figure 7d).

The results in Figures 3a and 7 show that increasing phase 2 repolarization (e.g., by using I_Ks_ enhancers) and decreasing phase 3 repolarization (e.g., by using I_Kr_ and I_K1_ blockers) results in a smaller APD percentage shortening (Figure 3a, top) with an increased stimulation rate, and in a robust positive rate dependence (Figure 3a, bottom).

### 3.4 Effect of action potential triangulation on the repolarization reserve

Interventions that prolong the action potential with a positive rate dependence result in a triangulation of the action potential (Figure 7). It has been postulated that triangulation of the action potential could be pro‐arrhythmic (Hondeghem et al., 2001; Kannankeril et al., 2010; Shah & Hondeghem, 2005). In this section we estimate how the triangulation of the action potential affects the repolarization reserve (an indicator of the pro‐arrhythmicity of an action potential) for the different interventions illustrated in Figures 4, 5, 6, and 7.

**FIGURE 8:**
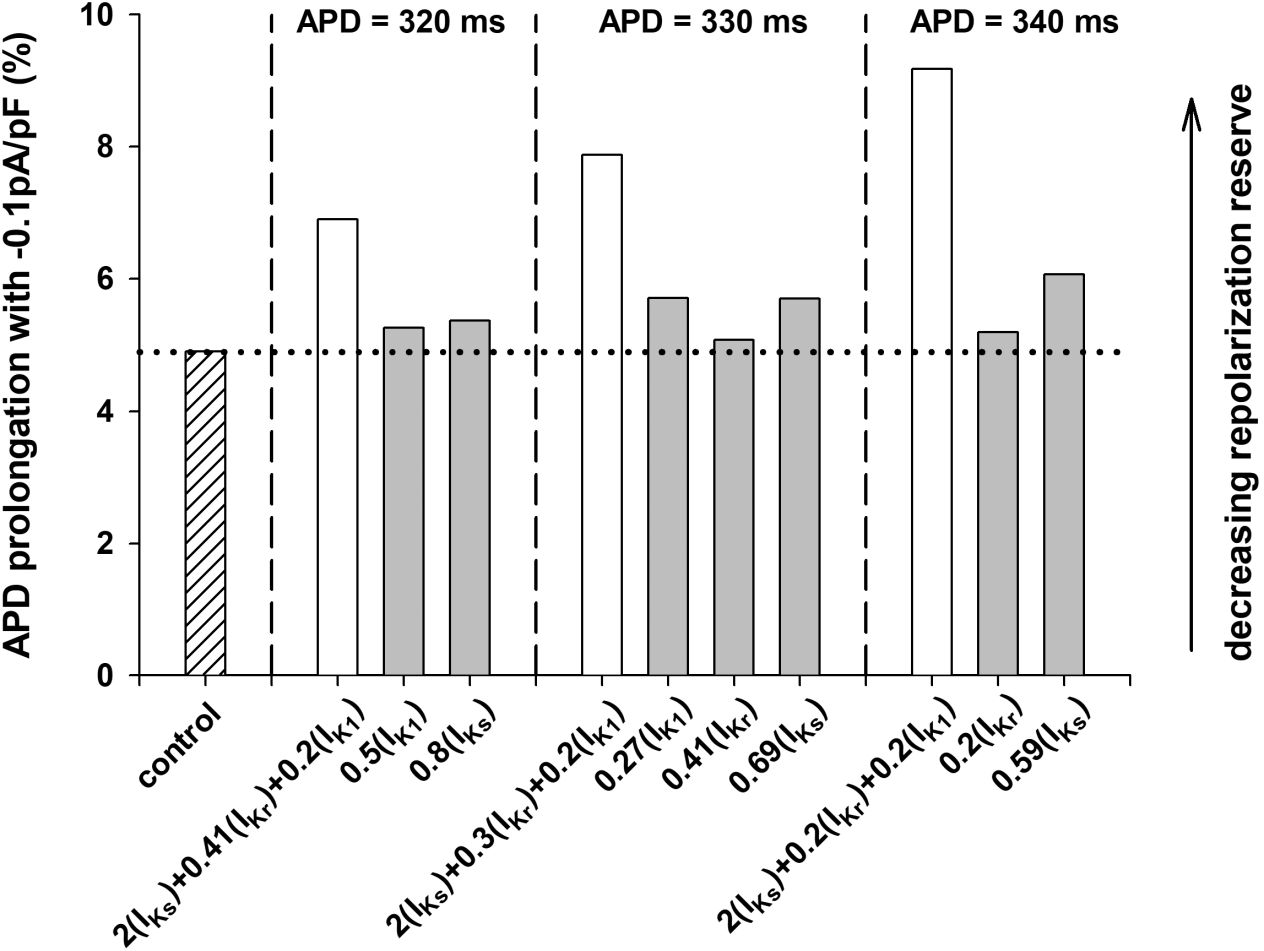
Percentage prolongation of the APD when a constant depolarizing current of −0.1pA/pF is applied during the action potential for control (bar with the hatched pattern), and the different interventions that result in APDs of 320, 330, and 340 ms at a BCL = 3000 ms (TNNP model). A larger APD prolongation indicates a decreasing repolarization reserve. White bars indicate interventions with a robust positive rate dependence (see Figure 3a, bottom). Gray bars indicate interventions with reverse or moderate positive rate dependence (see Figure 3a, bottom).

Figure 8 shows the percentage APD prolongation when a constant depolarizing current of −0.1 pA/pF is applied during the action potential for control (bar with the hatched pattern), and the different interventions that result in APDs of 320, 330, and 340 ms with BCL = 3000 ms. A larger APD prolongation with respect to the baseline APD indicates a smaller repolarization reserve. All interventions that prolong APD reduce the repolarization reserve when compared to control (horizontal dotted line in Figure 8). The interventions identified by the POS algorithm to prolong the APD with a positive rate dependence (white bars in Figure 8) decrease the repolarization reserve to a larger extent than interventions that prolong the APD with a reverse or moderate positive rate dependence (gray bars in Figure 8).

### 3.5 Effect of I_K1_ block on a positive rate dependence and the repolarization reserve

The substantial I_K1_ block (80%) for the interventions that result in a positive rate‐dependent response (white bars in Figure 8) likely contributes to the decrease in the repolarization reserve. We then investigated if it is possible to achieve APD prolongation with a positive rate dependence with more moderate levels of I_K1_ block.

**FIGURE 9:**
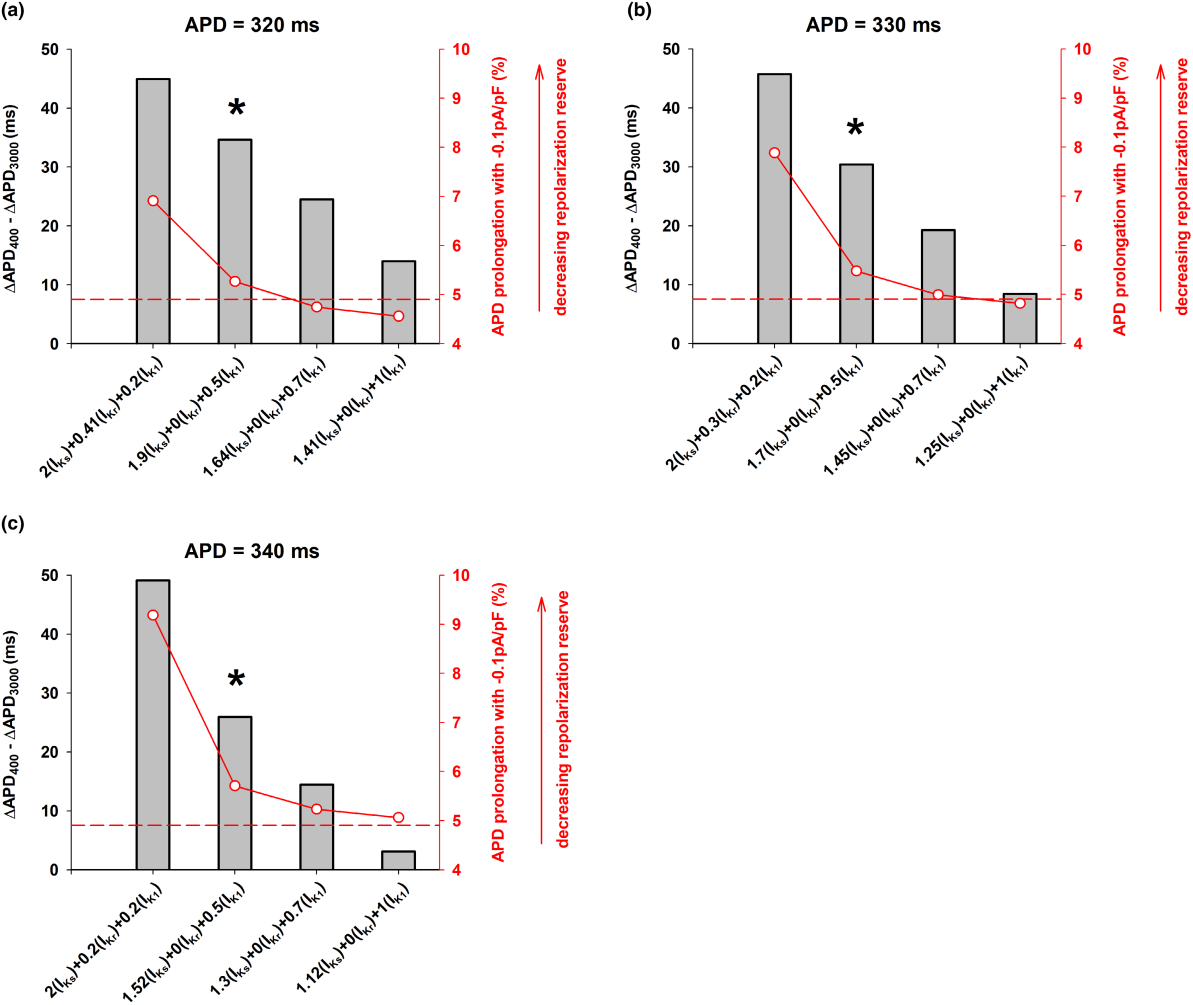
Interventions (x‐axis) that result in a positive rate dependence response (gray bars, left y‐axis) for different minimum values of I_K1_ block (80%, 50%, 30%, and 0%) in the POS optimization algorithm that result in APDs of 320 (panel a), 330 (panel b), and 340 ms (panel c) at a BCL = 3000 ms (TNNP model). The right y‐axis (red) shows the percentage prolongation of the APD when a constant depolarizing current of −0.1pA/pF is applied during the action potential. A larger APD prolongation indicates a decreasing repolarization reserve. The red horizontal dashed line shows the repolarization reserve for a control action potential. See text for detailed description.

The PSO optimization algorithm allows to constraint the range of variation of the maximum conductance of the different potassium channels (see Methods). Figure 9 shows the results of the PSO optimization algorithm when the lower limit of the range of variation of G_K1_ was 0.2×, 0.5×, 0.7×, and 1× the value of control. In all cases the upper limit of variation of G_K1_ was 2× the value of control. The range of variation of G_Ks_ and G_Kr_ remained between 0× and 2× the values of control.

Figure 9a shows different interventions that result in a APD prolongation to 320 ms with a positive rate dependence (gray bars, left y‐axis) for different levels of I_K1_ block (x‐axis). Figure 9a also shows an estimation of the repolarization reserve for the different interventions (red plot, right y‐axis). A higher value of the right y‐axis indicates a smaller repolarization reserve and vice versa. The results indicate, as expected, that lower levels of I_K1_ block result in a larger repolarization reserve (monotonic decrease of the red line plot in Figure 9a). The repolarization reserve when I_K1_ block is between 0% and 50% is about the same or below that of a control action potential (horizontal red dashed line). Figure 9a also shows that more moderate levels of I_K1_ block result in a decreasing positive rate dependence response.

Figure 9b and c show similar results for interventions that prolong APD to 330 and 340 ms, respectively. The strength of the positive rate dependence response and the repolarization reserve vary in different directions: a stronger positive rate dependence response results in a decreasing repolarization reserve and vice versa. However, most of the decrease in the repolarization reserve occurs for interventions that block I_K1_ by more than 50%. Interventions that limit I_K1_ block to 50% still result in a robust positive rate dependence response with a repolarization reserve similar to that of a control action potential (gray bars with an asterisk in Figure 9a–c).

## 4 DISCUSSION

We have shown that, in computer models of the ventricular action potential, APD prolongation by accelerating phase 2 repolarization (by increasing I_Ks_) and decelerating phase 3 repolarization (by blocking I_Kr_ and I_K1_) results in a robust positive rate dependence. In contrast, APD prolongation by blocking a specific potassium channel type results in reverse or a moderate positive rate dependence. Interventions that result in a strong positive rate dependence generally decrease the repolarization reserve because they require substantial I_K1_ block. Limiting I_K1_ block to ~50% could still result in a strong positive rate dependence with moderate decrease in repolarization reserve.

There is abundant experimental evidence showing that APD prolongation by blocking a unique potassium channel type results in reverse rate dependence (Banyasz et al., 2009; Barandi et al., 2010; Virag et al., 2009). Banyasz et al. (2009) and Barandi et al. (2010) showed consistent reverse rate dependence with several interventions causing lengthening and shortening of the action potential and concluded that reverse rate dependence is an intrinsic property of ventricular myocardium. Clinical results show that APD prolongation by blocking I_Kr_ in humans with different channel‐specific pharmacological agents also results in reverse rate dependence (Table 1 in Dorian & Newman, 2000). Thus, the available experimental and clinical evidence suggest that it is not possible to prolong APD with a positive rate dependence response by blocking a specific potassium channel type.

On the other hand, there is clinical and experimental evidence suggesting that it is possible to achieve APD prolongation with a neutral (neither reverse nor positive) rate dependence by modulating several ion channels, either by using one agent that interacts with different ion channels or by using several agents each one interacting with a specific ion channel type. For example, amiodarone, an agent that prolongs APD by blocking sodium and potassium channels, has an attenuated reverse rate‐dependent response when compared to agents that block selectively a unique ion channel type (Dorian & Newman, 2000; Hondeghem & Snyders, 1990). A reduction in reverse rate dependence can also be achieved using a combination of drugs affecting several channel types. For example, in an experimental model of the canine infarcted heart, the combination of esmolol, a short acting beta‐blocker that blocks I_K1_ and the L‐type Ca channel (Shibata et al., 2012), and d‐sotalol (an I_Kr_ blocker) prevented initiation of sustained ventricular tachycardia and attenuated the reverse rate dependence of d‐sotalol (Kassotis et al., 2003). It is possible that the mechanism for the absence of reverse rate dependence during treatment with esmolol and d‐sotalol is similar to the mechanism causing a positive rate dependence response in the computations presented here. Block of the L‐type Ca channel by esmolol (Shibata et al., 2012) could have resulted in an acceleration of phase 2 repolarization, and I_K1_ block by esmolol (Shibata et al., 2012) and I_Kr_ block by d‐sotalol (Tamargo et al., 2004) could have caused deceleration of phase 3 repolarization. Table 1 in Dorian and Newman (2000) summarizes the effects of class III antiarrhythmic drugs on rate dependence in humans: amiodarone and d‐l‐sotalol (combination of d‐sotalol and a beta‐blocker) show a neutral rate‐dependence, while different specific I_Kr_ blockers are all reverse rate‐dependent. Overall, these results suggest that the use of a combination of agents that affect various channels could be effective in achieving at least an attenuated reverse rate dependence with a consequent antiarrhythmic effect (Dorian & Newman, 2000; Hondeghem & Snyders, 1990).

In that context, we investigated optimal combinations of potassium channel activators and blockers that could result in a robust APD prolongation with positive rate dependence, and evaluated their potential pro‐arrhythmic risk. Our simulations, using two different computer models of the ventricular action potential (ten Tusscher et al., 2004; Tomek et al., 2019), consistently show that interventions that increase I_Ks_ and block I_Kr_ and I_K1_ prolong APD with positive rate dependence (Figures 2 and 3). But those interventions, which triangularize the action potential (Figure 7), could potentially decrease the repolarization reserve and be pro‐arrhythmic (Hondeghem et al., 2001; Kannankeril et al., 2010; Shah & Hondeghem, 2005). Figure 9 shows that interventions with higher levels of I_K1_ block (resulting in a more extreme action potential triangulation) lead to stronger positive rate dependence responses at the cost of a larger decrease in the repolarization reserve (Figure 9). However, interventions with moderate I_K1_ block (≤50%) still result in a robust positive rate response with moderate decrease in the repolarization reserve with respect to control (Figure 9, horizontal red dashed line). Those results indicating the importance of I_K1_ for a positive rate‐dependent APD prolongation are consistent with an earlier simulation study (Cummins et al., 2014) that identified G_K1_ as an important parameter to obtain a positive rate dependence. However, the experimental evidence on whether I_K1_ block results in positive or reverse rate dependence is inconclusive. Positive rate dependence has been demonstrated in guinea‐pig myocytes treated with terikalant (Williams et al., 1999), while block of I_K1_ with either terikalant (Biliczki et al., 2005) or BaCl_2_ (Virag et al., 2009) in canine myocytes results in reverse rate dependence. Interpretation of the experimental results is complicated because both terikalant and Ba^+2^ not only block I_K1_, but also block other potassium channels (Reilly & Eckhardt, 2021).

All interventions that result in a positive rate dependence response in our computations, for different amounts of APD prolongation and for different levels of I_K1_ block, require the use of I_Ks_ activators (Figure 9, x‐axis labels in panels A, B, and C). I_Ks_ activators have been developed to prevent excessive APD prolongation that may occur in patients suffering LQT syndrome, cardiac hypertrophy, or cardiac failure (Tamargo et al., 2004; Xu et al., 2002; Xu et al., 2015). I_Ks_ activators may also prevent excessive APD prolongation resulting from I_Kr_ and I_K1_ block in our simulations, and contribute to the modest decrease of the repolarization reserve for interventions with positive rate‐dependent response (Figure 9). All in all, it is possible that the use of a combination of I_Ks_ activators and I_Kr_ and I_K1_ blockers could result in APD prolongation that potentially maximizes antiarrhythmic effects (by maximizing APD prolongation at fast rates) and minimizes pro‐arrhythmic effects (by minimizing APD prolongation at slow rates) (Hondeghem & Snyders, 1990).

Several hypotheses have been proposed to explain the mechanism of reverse rate‐ dependent APD prolongation by blocking potassium channels (Barandi et al., 2010). Incomplete deactivation of I_Ks_ at fast rates could result in a repolarizing force that counteracts the effect of I_Kr_ block to prolong APD at fast rates (Jurkiewicz & Sanguinetti, 1993). It is also possible that an increase in the extracellular K^+^ concentration at fast rates could hinder the APD prolongation efficacy of I_Kr_ block at fast rates (Yang & Roden, 1996). Ion channels open and close during the cardiac cycle, and the interaction of ion channels and pharmacological agents may depend on the state (open or close) of the channel (Hondeghem & Katzung, 1977). During fast rates, myocardium is depolarized during most of the cardiac cycle (the opposite is true at slow rates). Therefore, if a pharmacological agent binds to the close state of the channel (i.e., during diastole) and unbinds when the channel is open (i.e., during systole), it will be less effective at fast rates than at slow rates, which would explain APD prolongation with reverse rate dependence.

Previous reports suggest that the shape of the action potential influences its rate dependence. The repolarization rate during late repolarization (i.e., the average I_ion_ current during phase 3 in this report, see Methods) can also affect the dynamics of I_Kr_ and I_K1_ (which in turn will also affect the repolarization rate) and have implications for reverse rate dependency (Virag et al., 2009). More recently, it has also been proposed that reverse rate dependence is an intrinsic property of ventricular myocardium resulting from the nonlinear relationship between the rate of repolarization (I_ion_) and APD (Banyasz et al., 2009; Barandi et al., 2010; Winter & Shattock, 2015). A consequence of that nonlinear relationship would be that baseline APD may be a determining factor contributing to reverse rate dependence (Barandi et al., 2010). Cummins et al. (2014) found a correlation between changes in action potential shape with stimulation rate and the potential for a positive rate‐dependent response by analyzing rate‐dependent effects of parameter perturbations in various ventricular action potential models. Our results showing that action potentials having the same APD but different shapes can have drastically different rate‐dependent responses (Figures 4, 5, 6 and 7) reinforce those earlier findings suggesting that the shape of the action potential influences its rate dependence.

### 4.1 Limitations

A number of factors should be considered when interpreting the results presented in this report. Computer models of the action potential have inherent limitations because model parameters are usually estimated from experimental data obtained under different conditions and from different preparations. Moreover, sometimes the experimental data needed to formulate the models is contradictory and/or incomplete. When compared to the ToR‐ORd model, the TNNP model has an unphysiologically large I_Ks_, which is a limitation. However, despite differences in ion channel density and kinetics between the TNNP and ToR‐ORd models, both models predict APD prolongation with a positive rate dependence for interventions that enhance I_Ks_ and block I_Kr_ and I_K1_ (Figures 2 and 3).

An increase in heart rate caused by beta‐adrenergic stimulation is accompanied by changes in the kinetics and conductances of important currents, including I_Ks_ and I_CaL_, and may significantly impact the effect of drugs at fast rates. The TNNP and ToR‐ORd models do not incorporate the effects of beta‐adrenergic stimulation on ionic channels, and, in our computations, faster heart rates were achieved by external stimulation. Therefore, the results of the simulations presented here do not account for the effects of beta‐adrenergic stimulation on ion channels and may not apply to the in‐situ heart.

The density and kinetics of ion channels of myocytes are typically altered by disease. For example, myocardial infarction leads to the remodeling of several ion currents (Cabo & Boyden, 2003), which increases the heterogeneity of ventricular myocardium and creates a substrate that makes it possible to initiate and sustain ventricular tachycardia (Baba et al., 2005). The effect of drug agents on remodeled myocytes may be different from their effect in healthy myocytes (Cabo & Boyden, 2003). Therefore, whether or not enhancing I_Ks_ and blocking I_Kr_ and I_K1_ will result in APD prolongation with a positive rate dependence in the remodeled myocardium that provides the substrate for ventricular tachycardia is unknown and deserves further study.

In addition to ion channel remodeling, gap junction conductance, and distribution is also remodeled by disease (Cabo et al., 2006). Therefore, even if APD prolongation with positive rate dependence in remodeled myocytes is possible, it is unknown whether that intervention will be effective in preventing initiation and/or maintenance of ventricular tachycardia in heterogeneous myocardium. Moreover, during action potential propagation, electrotonic load modulates the shape of the action potential and it can modify the effects of enhancing or blocking ion currents observed in single cells (Decker et al., 2009). Further computational and experimental studies will be needed to investigate how interventions that cause APD prolongation with positive rate dependence in single cells affect the dynamics of propagation of premature impulses that initiate and sustain reentrant arrhythmias.

## FUNDING INFORMATION

This work was supported in part by PSC‐CUNY Award # 65033–00 53

## CONFLICT OF INTEREST

The author declares that there is no conflict of interest.

## ETHICS STATEMENT

This study does not require ethical approval.
